# What is evidence as evidence is used? A case of dualism?

**DOI:** 10.1057/s41285-021-00170-4

**Published:** 2021-11-16

**Authors:** Andrew Neil Fletcher

**Affiliations:** Department of Philosophy, University of Durham, 50 Old Elvet, Durham, DH1 3HN UK

**Keywords:** Decision making, Deliberation, Evidence, Health service reconfiguration, Judicial review

## Abstract

How ‘evidence’ is conceptualised, generated and deployed in meso-level policy implementation on the ground is critical to health delivery. Using the case of a large-scale health service reconfiguration in northwest England, this study began as a narrative investigation into how different data types and sources are prioritised as NHS administrative structures change over time. During the research, one unpopular reconfiguration decision, the downgrading of a hospital, was challenged using judicial review. Suddenly, a key decision was being based not upon ‘facts and data’ type evidence but upon evidence of adherence to administrative procedure. This transferred focus away from the ever-shifting categories and hierarchies of data ‘types’ towards an emphasis on process. By comparing two deliberative contexts—committee and judicial review—this article proposes that evidence can be understood as simultaneously entity and process. As health service reconfigurations continue in response to austerity, integration agendas, evolving organisational landscapes, and demographic and political change, it is increasingly important to recognise the different meanings and uses of evidence.

## Introduction

Evidence, a master-term, has quite different meanings across contexts. This article will establish the crucial importance of ‘evidence’ terminology. It rehearses the key argument, that evidence takes divergent forms, using a case study of health service reconfiguration in Greater Manchester, UK, culminating in a judicial review (JR). The background and political context of the case study is described, followed by a detailed analysis. The article concludes with a warning, that in the context of EBP, we must exercise caution in our use of evidence terminology, which carries various political-contextual baggage. A more sophisticated understanding can inform policymakers, especially around long-term planning, and researchers examining how different forms of evidence are assembled to yield different policy outcomes.

The primacy of evidence in underpinning and justifying public policy decisions is broadly accepted as self-explanatory (Marston and Watts 2003, p. 144). Research on Evidence-Based Policy (EBP) has tended to emphasize the actual techniques used, giving rise to elaborate systematised processes of evidence generation and organisation, designed to convey fidelity to ‘scientific practice’. EBP has its origins in the logic of evidence-based medicine which, ‘…with its evidence hierarchies and emphasis on RCTs, meta-analyses and systematic reviews, sets the model for evidence-based policy almost everywhere’ (Cowen and Cartwright 2019, p. 1). While RCTs are still considered a ‘gold standard’ of evidence (ibid.: 7) in many contexts, their value is widely contested, especially in public policy (Cartwright and Hardie 2012). In fact, Byrne and Callaghan (2013) argue that the method is wholly useless for evaluating complex interventions in complex systems. In public health policy, where social determinants, values and context play significant roles, a broader array of evidence types is used. Here, especially at the intersections of health and social care, debates around how to manage such complex dynamic systems draw on a range of heterogeneous evidence types including ‘expert knowledge; published research; existing statistics; stakeholder consultations; previous policy evaluations; outcomes from consultations; costings of policy options; outputs from economic and statistical modelling’ (Cabinet Office 1999, p. 33). The tendency to categorise, assign values and place evidence ‘types’ into hierarchies (Weiss 2001; Brighton et al. 2003; Petticrew and Roberts 2003; Parkhurst and Abeysinghe 2016), attributes evidence with entity status—as data or material that functions to support a theory or argument, in this case, for making changes to the way health resources are allocated. But evidence hierarchies and health organisations are complex dynamic systems, adapting in relation to political or other contextual factors.

EBP has been criticised for being: over-reliant on methods such as RCTs (Cartwright and Hardie 2012); subject to replication problems (Begley and Ioannidis 2015; Saltelli and Funtowicz 2017; Camerer et al. 2018); overly technocratic and susceptible to political manipulation (Lewis 2003); undermined by the theory- and value-laden nature of facts (Reiss 2017); and unappreciative of the complexity of policy subjects and of policymaking itself (Cairney 2016). Recent approaches, such as EBM + in medical philosophy (Clarke et al. 2013, 2014) and complexity theories in social sciences (Castellani and Hafferty 2009; Byrne and Callaghan 2013; Holmes et al. 2016) enable more nuanced thinking about the meanings and uses of evidence in EBP. Here, context has become increasingly important in mainstream policymaking; for example, the Dutch Council for Health and Society operates a policy of ‘No evidence without context’, which advocates moving from evidence-based to context-based practices (2017). These developments have led to a softening in some policymaking circles from ‘evidence-based’ to ‘evidence-informed’, reopening both ontological and epistemological debates.^1^ Nevertheless, the debate and disagreement around EBP has created a context of perpetual critique; ‘the dirty secret [of EBP] is that we don’t know whether we’re doing the right thing’ (Guay 2018).

This article explores a departure from the idea of ‘entity’ evidence—a set of categorizable context-entangled informational ‘objects’—and proposes that evidence also comprises ‘processes’. This blended modality, incorporating both object and dynamic, offers a useful way to analyse how evidence operates in complex public administrative processes. In Greater Manchester, significant reconfiguration decisions were based on a range of evidence types, determined by and exposed to different organisational processes, audiences and forms of deliberation. The Greater Manchester context is described, including health service reconfigurations over the last decade and the present situation, known as ‘Devo Health’, which led among other things to the downgrading of a hospital, an event that became the subject of a legal challenge that treated evidence in a different way. This article explores aspects relating to evidence use in such decisions to support the thesis that evidence can be conceptualised equally well as an object, a process or both.

## The Greater Manchester case

Healthcare in the UK is mainly provided by the National Health Service (NHS). As a large and well-loved public institution, how the NHS performs and is organised is a matter of great public interest. This section describes two significant political factors affecting the NHS: (a) the UK government’s austerity programme and (b) the devolution of local health decisions in Greater Manchester.

Following the 2007/2008 global financial crash, the UK’s Conservative-Liberal Democrat coalition government initiated a programme of financial austerity in 2010. This involved reductions in public spending and selective tax increases.^2^ Although the government claimed that the NHS had been ‘ring fenced’ from such cuts, the austerity programme impacted all areas of public spending. For example, personal financial problems caused by welfare cuts led to increased mental health issues, placing further pressures on the NHS (Knapp 2012). Other complex relationships between health and social issues emerged, showing that the NHS remained vulnerable to the effects of austerity (Stuckler et al. 2017). The rate of growth for NHS funding almost flattened during austerity. “Budgets rose by 1.4 per cent each year on average (adjusting for inflation) between 2009/2010 and 2018/2019, compared to the 3.7% average rises since the NHS was established” (King’s Fund 2020). In Greater Manchester, decisions became increasingly based on financial rather than health-related factors, revealing austerity as a significant driver underpinning the reconfiguration decisions described here.

The widening funding gap, exacerbated by the UK’s growing and ageing population, resulted in the NHS having to make ‘efficiency savings’ of potentially up to £50bn by 2021/2022 (Roberts et al. 2012, p. 6). Overall, NHS organisational decisions have shifted in character from being clinically-based to economics-based (Horton 2017).

While this article focuses on Greater Manchester’s devolved health and social care context, the UK political structure remains highly centralized. Booth (2019) observes, “Not only is Britain a highly centralised state, it is constitutionally incoherent. In many cases, the government has devolved spending powers to Wales, Scotland and areas such as Manchester. However, it has not properly devolved tax-raising powers”. Consequently, devolved authorities such as Greater Manchester remain more accountable to Westminster than to their local populace. The Greater Manchester Combined Authority (GMCA) is therefore heavily reliant on central government funding allocations and so to wider (as well as local) political/financial factors.^3^

In 2011, Greater Manchester became a devolved ‘city-region’, governed by a combined authority. By 2015, the GMCA had been given increased control over its £6bn health and social care budget (then dubbed ‘Healthopolis’ but now known as ‘Devo Health’) and became responsible for the overall strategic direction of health and social care in Greater Manchester. This was seen as an opportunity to restructure services more in line with local needs and to pursue the NHS integration agenda as part of the broader narrative of health service reform. The present study began by examining the changing nature of evidence types used in various reconfigurations since 2005. During the research, one controversial decision, the downgrading of a hospital, was challenged using judicial review. This raised a different set of questions about the nature of evidence and its use, and became the new focus of the study.

### Evidence used in health reconfigurations in Greater Manchester since 2005

It is possible to describe a timeline of how different evidence types have been prioritised according to NHS organisational changes and wider political or economic drivers. The mid-2000s ‘Healthy Futures’ and ‘Making it Better’ reconfigurations across Greater Manchester were driven by four main arguments: a clinical case for the merits of concentrating services; a policy case—compliance with the European Working Time Directive; epidemiological evidence based on population projections; and a public consultation. These were approved by a joint committee of Primary Care Trusts (PCTs, NHS administrative bodies responsible for commissioning services). However, the evidence was strongly criticised and challenged by Ruane (2007), who disputed the quality/volume evidence that underpinned the ‘concentration of services’ argument; indicated key factors missing from the epidemiological evidence; and suggested that the way this was presented to the public was biased. Ruane’s argument was ignored by the Independent Reconfiguration Panel for NHS Service Change and the reconfigurations were implemented. This happened under a Labour government at a time of relative prosperity for public services, so while economic factors informed decision-making, these were less apparent in any rationale.

Supplanting PCTs, Clinical Commissioning Groups (CCGs) were established following the Health and Social Care Act (2012), enabling clinicians with better knowledge of local needs to take meso-level decisions around resource allocation and service configurations. GP Clinical Directors were required to run their practices as businesses, so began to take more corporate approaches, giving increased prominence to accounting evidence. A more business-focused NHS emerged into an austerity context; configuration decisions increasingly hinged on ‘doing more with less’. This is reflected in the NHS’ gradual move away from employing CIPFA (Chartered Institute of Public Finance and Accountancy) accountants, who pursue more traditional ‘book balancing’ approaches, towards CIMA (Chartered Institute of Management Accountants) accountants, who pursue more strategic and profit-oriented methods. There is also a trust issue; medics are perceived as ‘heroic’ (in comparison to accountants or politicians), so the idea of CCGs shaping local health economies carries a great deal of intuitive value in convincing members of the public in consultation. However, clinician-originated evidence may also be aimed at an internal audience, who want to be reassured they are making the right decisions (Byrne 2011, p. 109).

These structural and perceptual changes corresponded with the launch of Healthier Together, which was more explicitly finance-oriented. The overarching Devo Health strategy was required to save £2bn over a five-year period so the austerity mantra of ‘do more with less’ therefore provided a strong justification for service integration. Financial reasoning continues to determine NHS organisational structure. For example, ‘Sustainability and Transformation Partnerships’ (from which Greater Manchester is currently exempt), introduced in 2017, have been denounced as being explicitly financially motivated.

It is clear (to me at least) that the NHS is moving away from the legal framework embedded in the 2012 Health and Social Care Act which if my memory doesn’t fail me was based on competition between providers, guided by local commissioning led by local GP’s, and away from “political” decision making to one driven by market forces, subject to economic and quality regulation… Commissioning has moved (or is moving…) from a local base to one framed by the Sustainability and Transformation Partnership footprint (Steer 2019).

### Healthier Together, Devo Health and organisational discrepancy (2012-present)

‘Healthier Together’, launched February 2012, sought to rationalise healthcare provision across Greater Manchester by moving treatment out of hospitals and into community settings, enabling the former to provide better services and the latter to benefit from ‘joined up care’ between local councils and the NHS (Healthier Together 2015). This explicitly clinically-led programme is run by a ‘Committees in Common’ (CiC) of GP representatives from each of Greater Manchester’s 10 CCGs, amplifying the clinical voice and leveraging the commissioning power to implement new strategies alongside more centralised political and financial agendas (Heritage 2014). However, the programme became subsumed and heavily influenced by Greater Manchester’s wider devolution project and its inherently complex local political, spatial and economic factors (Checkland et al. 2015).

While not part of Healthier Together’s *original* remit, integrating health and social care is part of NHS England’s Five Year Forward View (2014), a planning document that set out to establish new models of care across seven domains (community, primary, emergency etc.), with an emphasis on serving local needs, as well as on collaboration and integration across a range of services including social care. This is considered a valuable strategy in terms of efficient use of resources, especially during a period of austerity. ‘Policy-makers and payers [sic] in both the public and private sectors place great hope in [integrated care’s] ability to save money…’ (Kodner and Spreeuwenberg 2002, p. 2). Despite Healthier Together claiming that its processes did not focus on wider financial sustainability (2015, p. 36), some stakeholders raised concerns that the proposed changes ‘were being driven primarily by financial considerations as opposed to a commitment to improve outcomes’ (ibid.: 44). While the aim of ‘joined up care’ may be underpinned by efficiency drivers, it is also clinically rational to co-locate or improve links between related services—and rationality was an important factor in the judicial review.

Devo Health refers to a number of significant changes that took place in the Greater Manchester health economy between 2012 and 2019. Both Healthier Together and Devo Health are overseen by the Health and Social Care Strategic Partnership Board (HSCSPB), whose stated remit is to progress the integration agenda by ‘finding what works on a local level, and… responding to what people need across all ten boroughs’ (2018). Having broad representation from across health, social care and emergency services enables a diverse array of evidence types to inform the board’s overall strategic direction, although the success of this enterprise requires a great deal of cooperation and collaboration. This leads to a problem; CCGs are purchasers, so Healthier Together, which holds statutory authority for local service delivery, operates in a *competitive* manner. But Devo Health, which has responsibility for the overall strategic direction of health and social care in GM, requires a more *collective* approach among the diverse representation of its governing HSCSPB.

In this administrative tangle, different evidence types have different values. Healthier Together strongly emphasised being clinically-led and responsive to a public consultation. Its decision-making was informed by both clinical and consultation evidence.^4^ However, it was bound by the strategic decisions of the HSCSPB, which drew on evidence from a wider pool of organisations and had less clear public representation. It remains unclear which of the two organisations—the CCG-led Healthier Together, or the more diverse but also more politically and financially-informed HSCSPB—had the ultimate decision-making power over ‘on the ground’ reconfiguration. Checkland et al. (2015) express this as: the organisational complexity that arises when a secondary healthcare system, based on competition in an internal market, has a strategic layer added to it that requires cooperation and coordination.

The Healthy Futures and Making it Better reconfigurations; the introduction of CCGs; Healthier Together and Devo Health all used different forms of entity evidence, determined by the prevailing NHS organisational structure at the time, to justify service reconfiguration decisions. While this is unsurprising, evidence can also be used in a different way…

### Judicial review (2015–2016)

It was determined in March 2015 ‘that there must be a “specialist” hospital in the southern sector’ (Healthier Together 2015, p. 169). Healthier Together’s ‘Decision Making Management Report’ (DMMR, which presented data and information to support the Healthier Together CiC in making its configuration decisions) identified a large postcode-defined catchment area, ‘South of GM’ (2015, pp. 130–131), in which Wythenshawe and Stepping Hill, being similarly sized general hospitals, were the only viable options for providing specialist care.^5^ Wythenshawe is a major acute teaching hospital (at the time, part of University Hospital of South Manchester NHS Foundation Trust, which served a population of approximately 570,000), with around 910 inpatient beds and providing district General Hospital services, as well as specialist tertiary and a range of medical and surgical acute services (Care Quality Commission 2016b). Stepping Hill is one of three hospitals run by Stockport NHS Foundation Trust, which serves a population of around 350,000. It is the Trust’s main acute site, providing a full range of hospital services including emergency care, critical care, and a comprehensive range of elective and non-elective general medicine and surgery. The hospital had 833 inpatient beds in 2016 (Care Quality Commission 2016a).^6^ The local area of Stockport has slightly less poverty and slightly better health than Wythenshawe (GMCA 2021).

In 2015, Healthier Together announced that Stepping Hill Hospital had been conferred specialist status in emergency abdominal surgery, resulting in that specialism being removed from—and effectively downgrading—Wythenshawe Hospital (despite Healthier Together initially claiming that no services would be removed or hospitals downgraded).

Clinicians from Wythenshawe Hospital mounted a legal challenge against the decision through judicial review (JR), a process of the administrative court that enables an individual or group to challenge decisions made by a public body. JR examines whether the process by which the original decision was made (a) followed the law and (b) was ‘rational’ (defined, according to Lord Diplock (1985), by its opposite, irrationality: ‘…a decision which is so outrageous in its defiance of logic or of accepted moral standards that no sensible person who had applied his mind to the question to be decided could have arrived at it’). However, JR does not generally decide whether the decision was good or bad for those affected. The clinicians claimed that some clinical outcomes had been ignored and therefore the consultation on which the decision had been based was procedurally flawed. The judge, Mr Justice Dove, found in favour of Healthier Together CiC on 7 January 2016. His full judgment, based around three core arguments, outlined below, provides an insight into the weighing of different evidence types and the process through which the decision was made:

1. *All hospitals will improve and past does not indicate future performance* Clinicians asserted that the CiC’s decision-making had ignored evidence from other hospitals’ past performances. Mr Justice Dove cited the ‘Decision Making Management Report’ (DMMR), which claimed ‘as current quality and safety standards will be improved in *all sites* [their emphasis], this should not be used as a criteria to determine the number of single services’ (2016, p. 122). By claiming that the reconfiguration would improve performance at all GM Hospitals, the CiC had pre-emptively downgraded the value of historic clinical data. Mr Justice Dove therefore rejected the assertion and was ‘satisfied that in principle it was *rational* for the defendant to put to one side existing data on clinical outcomes, on the basis that they are not necessarily a reliable basis for predicting clinical performance in the future…’ (2016: par. 108). Favouring local contemporaneous opinion over evidence on past performance acknowledges the complexity and dynamism of the system, indicating a conscious appreciation of context in relation to outcomes.
2. *Travel times* to A&E units across GM were calculated using computer models that had been calibrated against real data, providing ‘simulation’ evidence. The plaintiff claimed that the opening of a new link road in August 2017 had not been incorporated into the model, rendering the simulations invalid. Again, ruling in favour of the defendants—and of empirical realism—the Judge noted that adding ‘speculative’ (2016: par. 114) journey times into the model would have been inconsistent with the methodology of using actual data; ‘…an understanding of journey times derived from actual infrastructure on the ground, rather than infrastructure in the pipeline, was *not irrational*. It meant that the data used was transparent and could be checked, rather than being journey times which could only ever be the product of computer modelling’ (ibid.: par. 115). The claimant also cited ‘Hospital Episode Statistic’ data, a historic record of the relationship between travel times and clinical outcomes. The defendants reiterated that historical data provides no basis for future predictions, as it is limited to the ‘moment in time’ the data was extracted. Accepting the simulation evidence, irrespective of the new road, over historical clinical data indicates a judicial decision that respects, but is not in thrall to, clinical evidence.
3. *Co-location* refers to the planning of systems and pathways that cater for a range of conditions which rely on multiple interdependent services being available in the same place. Changing the availability of some services can affect other services outside the scope of the reconfiguration. It was claimed that the cardiovascular, vascular, burns and Cystic Fibrosis care provided at Wythenshawe would be potentially harmed by the loss of emergency and high-risk surgery on which they were co-dependent. However, a ‘post-consultation co-dependencies review’ (Healthier Together 2015: appendix 45) surveyed a range of literature relating to each area’s dependency and, while some of this evidence came from other bodies engaged in reconfiguration and was at best secondary, this satisfied Mr Justice Dove that the consultation had been carried out correctly. It was concluded that: for cardiovascular and Cystic Fibrosis services, although co-dependencies exist, co-location is not essential (Mr Justice Dove 2016: pars. 38 and 41); that specialist sites would require ‘clear and robust pathways’ to sites that provided vascular surgery (par. 39); and that specialised burns treatment is co-dependent with general surgery, available at University Hospital of South Manchester (par. 40).

In short no co-dependency issue was identified as marking out a preference for any particular hospital to be identified as the final remaining Specialist Hospital. Any outstanding issues could be addressed and accommodated after the selection had been made and as implementation occurred (par. 46).

In (1) and (2) the judge deemed past performance clinical outcome data and computer-modelled projection data to be not necessary for rational decision-making; and in (3) he validated the CiC’s reading of co-location data as rational in its aim to reach a decision. Critically, the judge was not able, nor required, to appraise the data itself; his role was to determine that there was a rational process at work. The key words in these arguments are: ‘rational’ (in the case of hospital improvement and travel times) and ‘carried out correctly’ (in the case of co-dependencies). These are verdicts on process, as opposed to specific entity evidence (a clinical opinion, a balanced book, an epidemiological prediction). The judge based his final decision upon how specific pieces of evidence were handled, rather than on a simple hierarchy of evidence types.

It is worth noting that several of the plaintiff’s key assertions were pre-empted or mitigated in the DMMR, which determined how specific pieces of evidence should be treated and what types of information were admissible into the decision-making process. The judge observed that the assumptions made for the report effectively ‘cast a form of invisibility cloak over the question of quality and safety and has the effect of excluding it exerting any form of influence over the final selection of the fourth Specialist Hospital’ (Mr Justice Dove 2016, par. 109). In other words, the DMMR provided a sort of ‘meta-evidence’ of process, which appeared to stem in advance assertions of procedural impropriety.

## Treatment of evidence in judicial review vs. committees

The judge’s report, in its description, deliberation and treatment of evidence from both sides, and its focus on providing an independent rationale, is arguably more transparent than many of the documents produced by Healthier Together and the wider Devo Health project. Although a judge lacks expertise in the complexities of health service configuration, the decision being made in a judicial review is different in the following ways:

- JR determines if *procedure*, assumed to be a rational process for the treatment of direct evidence, has been followed—it seeks evidence of *process*. A committee deliberates on the relative merits of direct evidence. *How* this is done becomes the evidence considered in judicial review, which may lead to a different outcome.
- JR considers if the challenged decision was *rational* (Mr Justice Dove’s report contained 25 references to ir/rationality). The rationality of a committee is to some extent determined by its wider organisational context but also by its adherence to ‘logic’ and ‘accepted moral standards’ (Lord Fraser et al. 1985). Arguably, the JR in this case had to consider each of these three slightly different angles.
- The decision-making power of a group—albeit an unevenly balanced group of experts in different fields, subject to multiple external influences—is delegated into the hands of a single (theoretically more ‘objective’) expert in law and administrative procedure.

This change in the treatment of evidence marks a significant turning point for the democratic deliberation of public policy. Despite being a well-established resource, designed to ensure the separation of powers in modern governance, some believe JR is becoming too heavily relied upon. Dyson (2015, pp. 2–3) believes the ‘massive increase in the number of applications for judicial review’ is due to (1) the standard of review becoming more relaxed; (2) an ‘explosion of [rushed] legislation’, generating uncertainty and litigation; and (3) national and international challenges causing public bodies to take more risks. Theoretical debates around the role of JR in what is deemed the ‘democratic ontology’ (Lustig and Weiler 2018, p. 316) remain open. Recourse to a comparatively objective process can bring some order to the problems of complexity and evidence amalgamation that affect meso-level decisions by committee around health and social care provision. This has implications for the types of evidence that are sought, recorded and used by public bodies, and for the processes through which such decisions are made.

## Nature and deliberation of evidence

To return to the original discussion of the *nature* of evidence—as entity, process, or some dual version of both—the developments in Greater Manchester suggest that it is best understood precisely in terms of the interaction between both *in context*. The positivist style of insistence that evidence is something known which should guide action, a key principle of those who argue for hierarchies of evidence with the most scientific (RCTs) having the most weight, never describes how evidence is actually deployed in any real social context. This is probably true of many apparently clinical deployments but it is necessarily true of any policy formation and implementation process that involves the interaction, or interweaving, of complex health, social care, political, and urban systems within a dynamic fiscal context in which resources are massively constrained. In their critique of an Institute for Public Policy Research (IPPR) report that purported to present evidence for hospital reconfiguration, Byrne and Ruane (2007) outline much the same argument, concluding that the IPPR evidence failed to acknowledge major contextual factors around interdependency, the volume/outcome relationship and patient empowerment. The IPPR report was further undermined by being partially sponsored by a commercial organisation.

Opinions around the best ways to organise democratic deliberation vary. Landemore (2017) believes in collective judgment, the need for large diverse groups, as objectivity can only be pursued through the mixing of equally weighted different perspectives, whereas Somin (2014) says that big groups are inefficient and result in the dilution of knowledge, and smaller groups of experts (subsidiarity) are better. On epistemic diversity, a distinction is made between ‘teams of experts’ (who operate independently) and ‘expert teams’ (whose combined expertise is greater than the set of individuals), and their relative merits in deliberating complex issues (Burke et al. 2004; Salas et al. 2006; Reyes and Salas 2019). This is of particular importance in integrated health and social care contexts, where problems such as ‘epistemic injustice’—treating people differently because of the type of knowledge they hold (Fricker 2007)—can emerge. This mainly philosophical concept has special relevance in healthcare and health policy, where it can lead to unequal power relationships between agents as sources of evidence. For example, in healthcare, where multiple professions with different statuses work together (increasingly as services become more integrated), ‘clinical knowledge’ is often prioritised over other forms of knowledge (Fletcher and Clarke 2020), whereas in a business context, financial evidence often carries more weight.

Healthier Together and Devo Health relied on deliberation among differently sized and differently constituted groups. Healthier Together was led by a small group of clinician-managers (an expert team) and organised around a public consultation (then later tied to the wider politics of Devo Health), whereas Devo Health involved a large group from a range of public services (a team of experts). Both processes relied on cooperation at various levels and on the deployment, weighing and organising of different types of entity evidence. Using a variety of heterogeneous evidence types to calculate the best course of action from a range of options leads to more robust conclusions (Fletcher et al. 2018). But external influences, political and financial, shape the discussion, while internal influences such as rhetoric and internecine competition drive that discussion in certain directions. These factors determine which evidence types are admitted, how they are valued relative to one another, and how their outcomes will be perceived more widely.

Judicial review is, theoretically, less vulnerable to the wider influences of state, public opinion and other threats to objectivity. Where committees amalgamate evidences as separate entities, JR considers that amalgamation process itself. It is a model of contestation, not deliberation; an adversarial process in which two opposing parties present evidence of whether due process has been followed or not. A single decision-making agent, the judge, then determines which argument to uphold based on his or her own expertise in law. The judge’s report offers some transparency—setting out the evidence from both sides and giving a clear rationale for the decision. This legal realist (as opposed to legal formalist) perspective reflects a view that those making legal decisions should pursue value-free approaches, and that the law does and should serve social ends, so legal decisions consider fairness and public policy—the *real* world as it is, rather than the more abstract world of rules (Tamanaha 2008). It has been said that legal realists ‘promoted the application of social science to law’ (Tamanaha 2016, p. 147). While committees evaluate the weight and relevance of different forms of entity evidence according to shifting hierarchies, JR treats different forms of entity evidence more equally and focuses instead on how these were processed. Each approach has its virtues but also highlights the slippery nature of the term ‘evidence’.

## Simultaneity

This article argues that the term ‘evidence’, as deployed in debates around evidence-based policy, should be used with more caution and more nuance. Specifically, evidence does not solely refer to ‘facts and data’ (which seem condemned to jockey for position within an artificial hierarchy determined by prevailing organisational and political contexts); it can also be understood as the *process* through which facts and data are put—the basis for judicial reviews. The idea that evidence might exist simultaneously as both entity and process makes a useful contribution to discussions around evidence-based policy. This simultaneity is important; a more dynamic and process-oriented understanding of evidence improves on the somewhat more static concept of categorical and hierarchical evidence ‘types’, which are ‘profoundly unpragmatic’ (Byrne 2011, p. 46). Just as causal mechanisms do not provide complete explanations without some understanding of their context, entity evidence must be considered in tandem with the process it is entered into. In complex meso-level decisions, such as those concerning health service reconfigurations or integrating health and social care services, we already recognise that context plays a vital role. It should not be too great a leap to consider evidence in similar terms.

## Conclusion

The use of JR in health service configuration decisions mobilizes a different set of background understandings around evidence, in which evidence must be considered as both an entity *and* as a process determining an outcome. While the distinct disciplinary forms of facts/data-type evidence move in and out of alignment with the shifting priorities of different NHS structures, the JR focused solely on the logic and administrative process of the original committee decision. The shift from using ‘disciplinary-based’ evidence, with fluctuating values linked to a prevailing culture, to being an instrumental tool in the service of a ‘higher’ decision, reflects this shift in focus from object to process. As judicial reviews become an ever more popular way to contest unpopular administrative decisions, the idea of evidence as a dynamic complex becomes increasingly relevant.

This more sophisticated understanding can contribute to the thinking underpinning health service reconfigurations and other EBP-related decisions. First, we might consider those deploying evidence in committee-style decision-making contexts as skilled builders, assembling institutional-policy life from different levels and forms of evidence. Accounting for the wider context, the process of admitting and considering different evidence types, might enable decisions that are more resilient to organisational and contextual vagaries. At the very least, knowing that the administrative process itself might become evidence in a final decision should inform strategies at the committee stage. Second, for those studying related topics, such as knowledge aggregation, better recognising the relationship between facts and the process they are entered into might enable new insights in the more abstract debates around EBP. Either way, the core message remains: evidence terminology is slippery and more nuanced understandings of it would significantly benefit complex decision-making, especially in health and broader public policy.

## Footnotes

1 Ontological: is evidence a thing or a process, or both? RCTs and other scientistic approaches assume a mechanistic ontology to reveal the true nature of things. Epistemological: evidence is how we justify the belief in an action, so what is our relationship to it? ‘Justification’ is itself highly value-laden and the use of evidence is instrumental in our own value systems. In the political realm, ideas like ‘facts’ and ‘truth’ have only partial currency.

2 Selective tax increases: for example, the rate of value-added tax, a regressive tax, was increased, while the marginal tax rate on the highest incomes was reduced.

3 A discrepancy recently illustrated (October 2020) by the government imposing Covid-19 lockdown restrictions on Manchester while limiting its financial support to the city.

4 Such consultations often involve presenting a clinical argument to the public to inform its opinion so it is debatable how much these two evidence sources might offer usefully different perspectives.

5 Tameside General Hospital and Macclesfield General Hospital (to the east and south of GM respectively) were also in scope during the early planning stages but fell outside of the DMMR catchment area.

6 This comparison uses 2016 data, from closer to the time of the original specialist status decision in 2015. Wythenshawe Hospital has been part of Manchester University NHS Foundation Trust since 1 October 2017, which serves a larger population of 750,000.
